# Supplementary material for: Status of Ecosystem Services in Abandoned Mining Areas in the Iberian Peninsula: Management Proposal
Source: Toxics. 2023 Mar 17;11(3):275. doi: 10.3390/toxics11030275 (PMC10051972; doi:10.3390/toxics11030275)
Supplement: Supplementary file 1 [file toxics-11-00275-s001.zip › toxics-2273186-supplementary.pdf]

Toxics

Supplementary Materials for

Status of Ecosystem Services in Abandoned Mining Areas in the Iberian Peninsula: Management Proposal

María González-Morales, Mª Ángeles Rodríguez-González and Luis Fernández-Pozo *

Environmental Resources Analysis (ARAM) Research Group, University of Extremadura, 06006 Badajoz, Spain
* Correspondence: luferpo@unex.es

Contents of this file

1. **Figures**

Figure S1. Mining tailing platform.

Figure S2. Mining tailing.

Figure S3. Buildings.

Figure S4. Dump.

Figure S5. Rotated component chart.

2. **Tables**

Table S1. Diseases and/or disorders caused by metal(loid)s in humans.

Table S2. Soil analysis.

Table S3. Correlation matrix.

Table S4. Component matrix.

Table S5. Component matrix rotated.

1. Figures

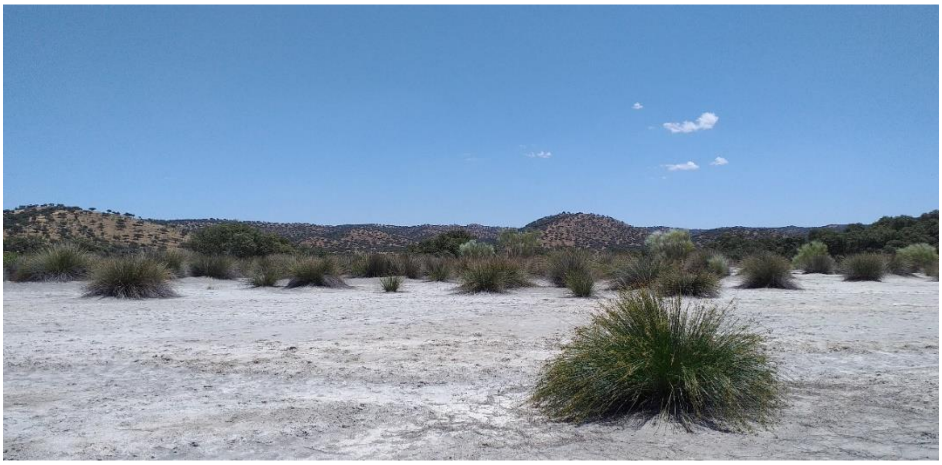

Figure S1. Mining sludge platform.

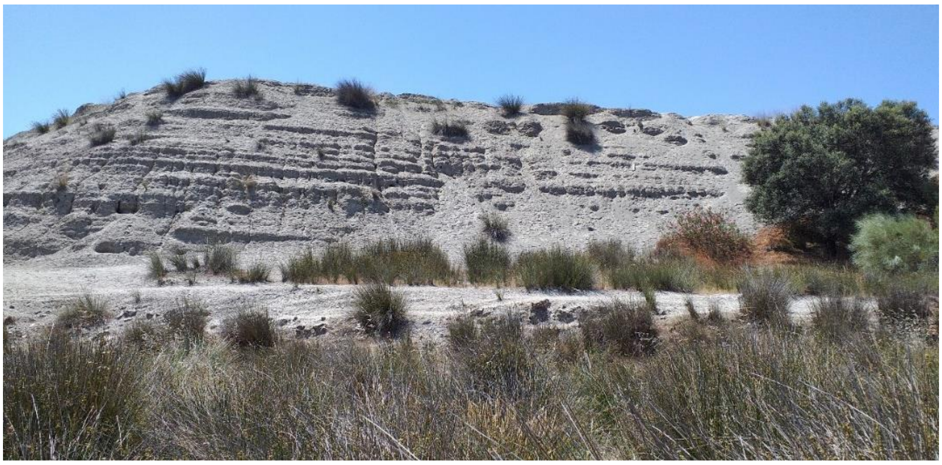

Figure S2. Mining sludge.

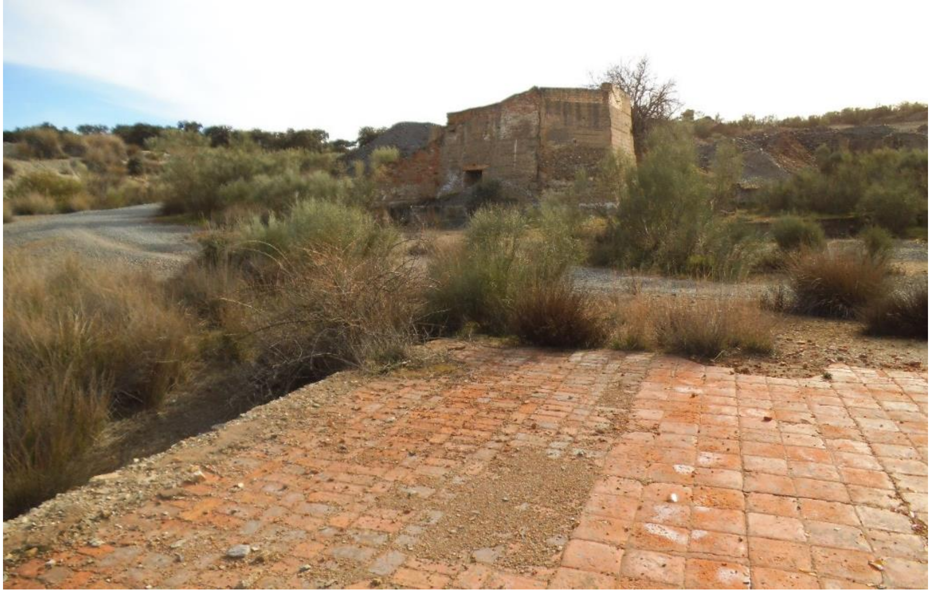

Figure S3. Buildings.

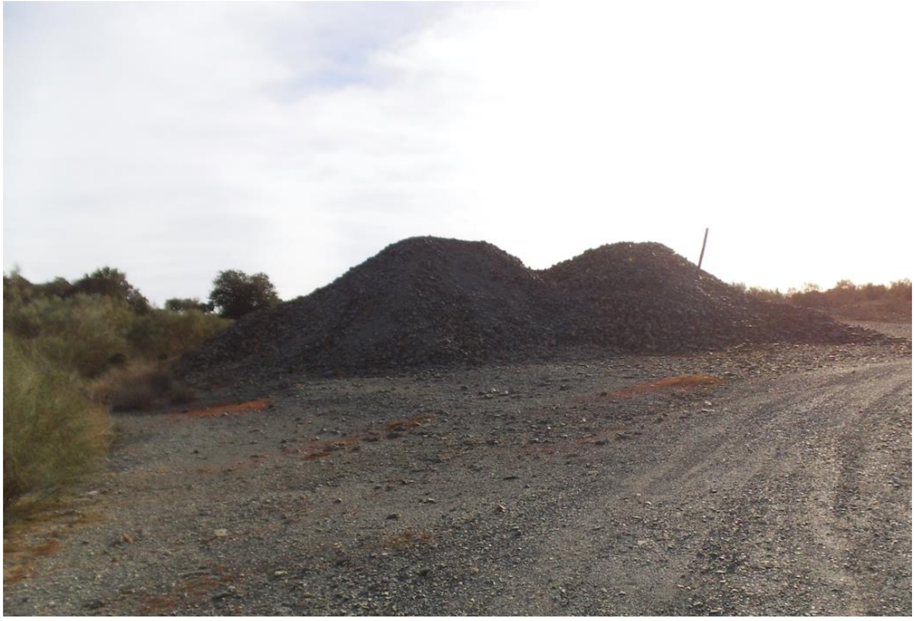

Figure S4. Dump.

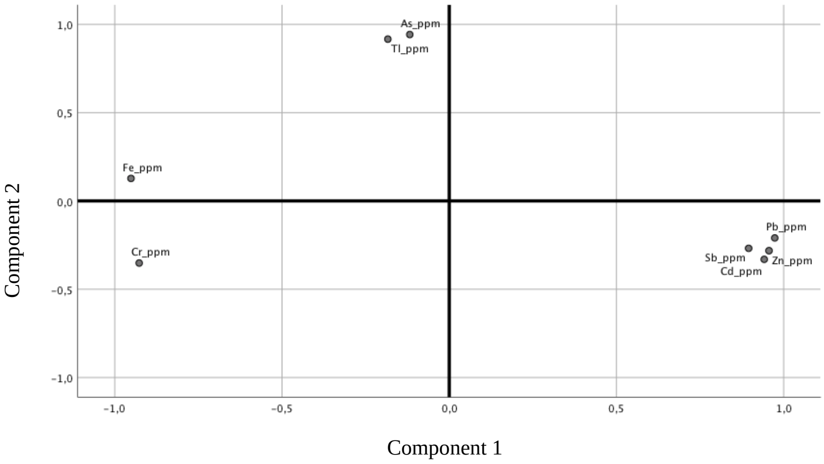

Figure S5. Rotated component chart.

2. Tables

Table S1. Diseases and/or disorders caused by metal(loid)s in humans.

Metal(loid)	Diseases/Disorders	References
As	Cardiovascular and respiratory diseases, tumor processes, immune dysfunction, liver disorders.	[16]
Cd	Respiratory, gastrointestinal diseases, kidney and bone disorders, potentially tumorous.	[17]
Co	Cardiovascular, pulmonary, endocrine, neurological, potentially tumor diseases.	[18]
Cr	Digestive tract ulceration, allergic reactions, tumor processes.	[19]
Hg	It affects the nervous system, respiratory diseases, cardiac and gastric alterations.	[20]
Ni	Epithelial irritations and inflammations, pulmonary, haematological, renal and gastric diseases, tumor processes.	[21]
Pb	Haematological, neuronal, skeletal dis9eases, development in children, tumor processes.	[22]
Sb	Respiratory, cardiovascular and/or gastrointestinal diseases.	[23]
Se	Respiratory, cardiovascular, gastrointestinal and/or motor diseases.	[24]
Tl	Affects lungs, kidneys, heart, liver, reproductive disorders and nervous system.	[25]
Zn	Respiratory problems, digestive tract disorders, behavioural disturbances; tumor processes.	[26]

Table S2. Soil analysis.

	Eh (mV)	Ca (Cmol/kg)	Mg (Cmol/kg)	Na (Cmol/kg)	K (Cmol/kg)	Arena (%)	Arcilla (%)	Limo (%)
Mining sludge	318.2 ±7.7	2.33 ±0.71	0.71 ±0.36	0.22 ±0.9	0.21 ±0.04	35.21 ±12.07	7.54 ±1.77	57.25 ±10.53
Shrubland	326.9 ±4.9	4.60 ±1.44	1.12 ±0.46	0.17 ±0.07	0.26 ±0.09	47.36 ±4.66	7.53 ±1.35	45.11 ±4.36
Riparian	303.8 ±47.0	6.57 ±2.38	1.80± 0.82	0.17 ±0.07	0.31 ±0.13	55.97 ±11.69	4.96 ±2.12	39.07 ±10.65
Dehesa	340.2 ±61.5	5.62 ±2.01	1.63 ±0.76	0.20 ±0.09	0.27 ±0.12	56.49 ±8.94	7.85 ±2.13	35.66 ±9.44
Control	220.1 ±32.5	9.26 ±1.39	2.79 ±0.68	0.09 ±0.01	0.15 ±0.03	73.95 ±1.89	8.75 ±0.65	17.30 ±1.16

Table S3. Correlation matrix [a,b]

	**As**	**Pb**	**Sb**	**Tl**	**Zn**	**Cr**	**Fe**	**Cd**
**As**	1,000	-,340	-,404	,781	-,363	-,210	,166	-,431
**Pb**	-,340	1,000	,949	-,338	,983	-,829	-,929	,990
**Sb**	-,404	,949	1,000	-,321	,904	-,701	-,797	,953
**Tl**	,781	-,338	-,321	1,000	-,458	-,149	,385	-,461
**Zn**	-,363	,983	,904	-,458	1,000	-,793	-,968	,989
**Cr**	-,210	-,829	-,701	-,149	-,793	1,000	,851	-,751
**Fe**	,166	-,929	-,797	,385	-,968	,851	1,000	,923
**Cd**	,431	,990	,953	,461	,989	,751	,923	1,000

a. Determinant = ,000

b. This matrix is not certain positive

Table S4. Component matrix [a]

	Component	
	**1**	**2**
**Cd**	,998	
**Zn**	,997	
**Pb**	,992	
**Fe**	-,947	-,161
**Sb**	,934	
**Cr**	-,781	-,611
**As**	-,393	,865
**Tl**	-,448	,820

Extraction method: Principal Component Analysis

a. 2 components extracted.

Table S5. Component matrix rotated.

	Component	
	**1**	**2**
**Pb**	,973	-,210
**Zn**	,956	-,282
**Fe**	-,952	,128
**Cd**	,942	-,331
**Cr**	-,927	-,351
**Sb**	,895	-,268
**As**	-,118	,942
**Tl**	-,184	,916

Extraction method: Principal Component Analysis

Rotation method: Varimax with Kaiser normalization

a. The rotation converged in 3 iterations.
